# High risk of deep vein thrombosis associated with peripherally inserted central catheters in lymphoma

**DOI:** 10.18632/oncotarget.8639

**Published:** 2016-04-07

**Authors:** Xi Zhang, Jia-Jia Huang, Yi Xia, Chao-Feng Li, Yu Wang, Pan-Pan Liu, Xi-Wen Bi, Peng Sun, Tong-Yu Lin, Wen-Qi Jiang, Zhi-Ming Li

**Affiliations:** ^1^ State Key Laboratory of Oncology in South China, Collaborative Innovation Center for Cancer Medicine, Department of Medical Oncology, Sun Yat-sen University Cancer Center, 510060 Guangzhou, China; ^2^ Department of Information, Sun Yat-sen University Cancer Center, 510060 Guangzhou, China

**Keywords:** peripherally inserted central venous catheter (PICC), thrombosis, lymphoma

## Abstract

Peripherally inserted central venous catheters (PICCs) are widely used in cancer patients. Although PICC is a convenient tool, its use is associated with an obvious increase in the incidence of venous thrombosis. The risk factors for deep vein thrombosis associated with the use of PICCs in cancer patients are largely unexplored. This study aimed to investigate the incidence of PICC-associated thrombosis in lymphoma compared with its incidences in other types of cancer. A total of 8028 adult cancer patients inserted with PICC between June 2007 and June 2015 were included in this study. A total of 249 of the 8028 included patients (3.1%) inserted with PICC developed upper extremity deep vein thrombosis (PICC-UEDVT). Patients with lymphoma were more likely to have PICC-UEDVT than those with other types of malignancies (7.1% vs. 2.80%; *P* < 0.001). Logistic analysis revealed that a lymphoma diagnosis was a risk factor for UEDVT in cancer patients inserted with PICC (OR: 3.849, 95% CI: 2.334–6.347). Patients with lymphoma may be more predisposed to developing PICC-UEDVT than those with other types of malignancies. Identifying the mechanism underlying the relationship between PICC-UEDVT and lymphoma requires further study.

## INTRODUCTION

Peripherally inserted central venous catheters (PICCs) are vascular access devices that are inserted through a peripheral vein in the arm. PICCs are often used to deliver special medications, such as parenteral nutrition, intravenous antimicrobials and anti-carcinogens. They are also used to maintain venous access in patients who may require frequent phlebotomies, continuous medication administration and extended courses of chemotherapy [1]. Therefore, PICC is a convenient tool for use in patients with cancer, with benefits to both outpatients and inpatients.

PICC use has increased because of its many applications, ease of insertion, perceived safety, and cost-effectiveness compared with alternative venous catheters [2, 3]; however, its use also carries risks. Among the early and delayed complications associated with PICCs, the most notable is thrombosis. Thrombosis can complicate and interrupt treatment, in addition to increasing costs, morbidity, and even mortality [4]. Venous thromboembolism (VTE) is the second leading cause of death in cancer patients [5]. PICCs are strongly associated with the risk of developing upper extremity deep vein thrombosis (UEDVT) [6, 7], and patients with cancer, especially those with hematological malignancy [8], are at a high risk of developing VTE [9].

To reduce the risk of PICC-associated UEDVT (PICC-UEDVT), the factors contributing to this adverse event have been explored; however, they remain unclear. Previous studies have found that patient characteristics, treatments and catheter types are potential risk factors for PICC-UEDVT [10–13]. There may also be synergistic activity among these risk factors [14]. At our institute, we have observed that once a PICC is inserted, patients with lymphoma are more likely to develop UEDVT than those with another type of cancer. Therefore, the aim of this study was to compare the incidence of PICC-UEDVT between patients with lymphoma and those with other types of cancer.

## RESULTS

Between June 1st, 2007 and June 30th, 2015, a total of 9290 PICCs were inserted into patients at the Sun Yat-sen University Cancer Center. Of these patients, 41 were eventually diagnosed with benign disease, 525 were outpatients, 126 were less than 18 years of age, and 570 had incomplete data or failed to meet the inclusion criteria. Ultimately, 8028 patients were included in this study.

The median age of the included patients was 52, and the sex ratio was 1.46 (4771 males to 3257 females). Among these patients, 3536 had nasopharyngeal carcinoma or head and neck cancer (44.05%), 1916 had a gastrointestinal malignancy (23.87%), 1053 had breast cancer (13.12%), 565 had lymphoma (7.04%), 338 had reproductive system cancer (4.21%), 330 had lung cancer (4.11%) and 290 had another type of rare tumor (including 68 with a nervous system tumor, 48 with cancer of an unknown primary site, 37 with sarcoma, 29 with multiple myeloma, 27 with leukemia, 23 with thyroid carcinoma, 22 with a urinary system tumor, 20 with melanoma, 5 with thymic carcinoma, 3 with mesothelioma, 2 with malignant hemangioendothelioma, 1 with pheochromocytoma, 1 with hemangiopericytoma, and 3 with cutaneous carcinoma, accounting for 3.61% of all patients).

A total of 249 of the 8028 (3.1%) patients were diagnosed with PICC-UEDVT by Doppler ultrasound. Of them, 116 patients were younger than 52 years of age, and 133 were older than or equal to 52 years of age, and these numbers were 4084 and 3695, respectively, for the patients without UEDVT (*P* = 0.057). The range of the interval between the placement of peripherally inserted central catheters and the diagnosis of deep venous thrombosis was 1 to 331 days, and the median of the interval was 25 days. The median platelet counts at first visit and at the time of catheter insertion in the UEDVT patients were 180 × 10^9^/L and 207 × 10^9^/L, respectively, and these numbers were 200 × 10^9^/L, 166 × 10^9^/L in the patients without UEDVT. These values are all within the normal range. The platelet count at first visit was lower in the UEDVT patients (*P* = 0.032). However, there was no significant relationship between UEDVT and the platelet count at the time of catheter insertion (*P* = 0.089). With regard to the medical history of the UEDVT patients, 33 (13.3%) of the patients underwent surgery at > 1 hour before PICC insertion, 99 (39.8%) had a history of radiotherapy, and 211 (32.5%) had received chemotherapy through the PICC. The number of patients with a history of prior surgery did not significantly differ between the patients with and without UEDVT (*P* = 0.152); however, radiotherapy (*P* < 0.001) and chemotherapy (*P* = 0.0042) were positively correlated with UEDVT. With regard to the gender ratio, 153 (61.4%) of the patients with UEDVT and 4618 (59.4%) of those without UEDVT were male, indicating that was no significant difference in the incidence of UEDVT according to gender (*P* = 0.510). The proportion of patients with UEDVT among the cancer patients inserted with PICC differed according to cancer types. UEDVT tended to occur in the patients with lymphoma more frequently than in those with other type of cancers (*P* < 0.001), as shown in Table 1.

**Table 1:** Characteristics of cancer patients inserted with PICC stratified according to the presence or absence of UDEVT

Characteristic	UDEVT *n* = 249 (100%)	No UDEVT *n* = 7779 (100%)	*P*-value
Age (years)			
< 52	116 (46.6)	4084 (52.5)	NS^b^
≥ 52	133 (53.4)	3695 (47.5)	
Gender			
Male	153 (61.4)	4618 (59.4)	NS^b^
Female	96 (38.6)	3161 (40.6)	
Prior surgery (> 1 hour)			NS^b^
Yes	33 (13.3)	1303 (16.6)	
No	216 (86.7)	6476 (83.4)	
Radiotherapy			< 0.001^b^
Yes	99 (41.4)	3897 (50.1)	
No	150 (58.6)	3882 (49.9)	
Chemotherapy			0.042^b^
Yes	211 (85.1)	6938 (89.2)	
No	38 (14.9)	841 (10.8)	
Platelet at first visit	Median 180.0 × 10^9^/L	Median 200.9 × 10^9^/L	0.032^a^
Platelet at catheter insertion	Median 207.6 × 10^9^/L	Median 166.0 × 10^9^/L	NS^a^
Diagnosis			
Lymphoma	40 (16.1)	525 (6.7)	< 0.001^b^
Others	209 (83.9)	7254 (93.2)	

a Independent sample *t*-test.

b Chi-square test.

NS, not significant (*P* > 0.05).

PICC, peripherally inserted central venous catheter; UEDVT, upper extremity deep vein thrombosis.

**Figure 1:**
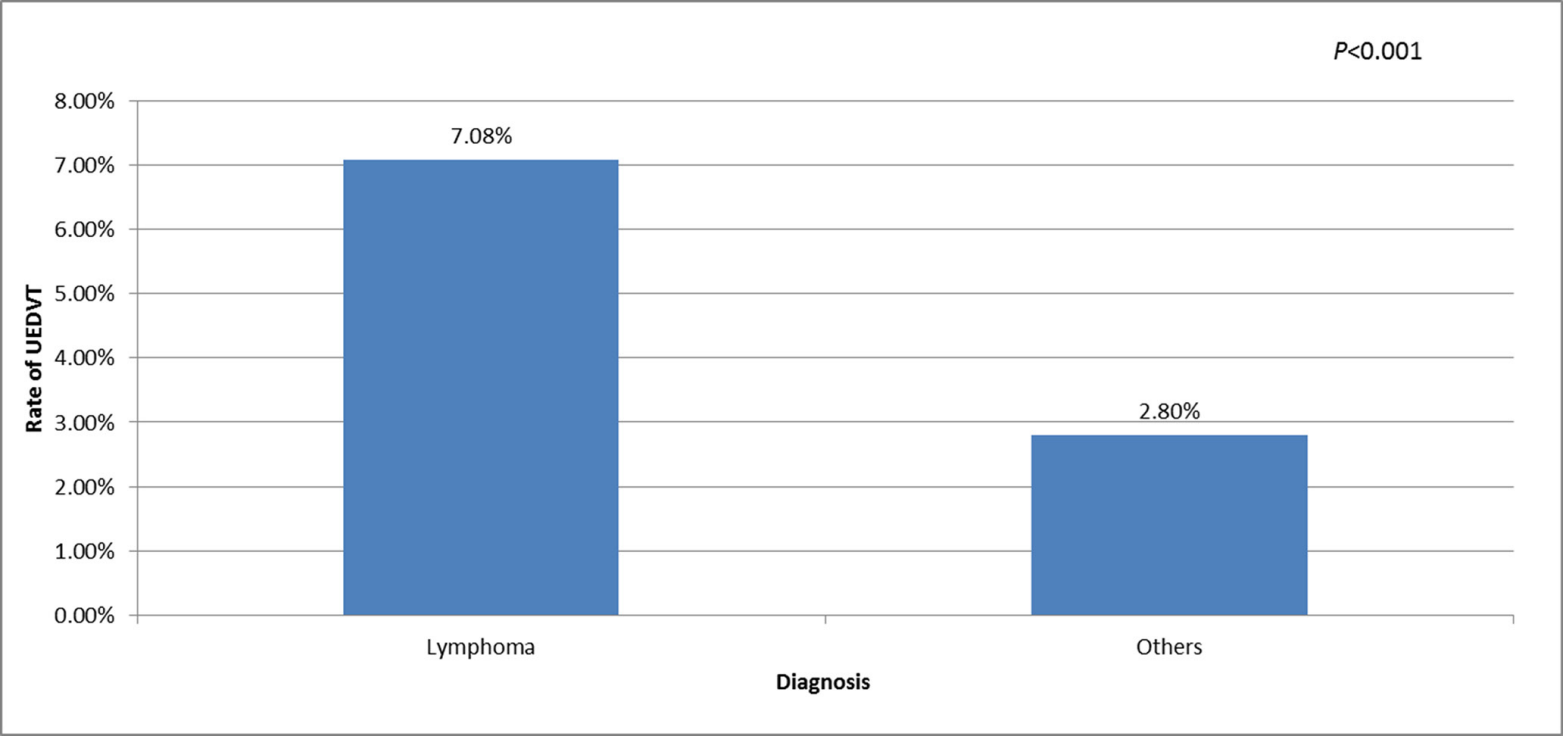
Rates of UDEVT in patients with lymphoma and those with other types of cancer UEDVT, upper extremity deep vein thrombosis; Others, nasopharyngeal carcinoma or head and neck cancer, gastrointestinal malignancy, breast cancer, reproductive system cancer and rare tumor.

In our study, 565 patients were diagnosed with lymphoma. Of them, 341 (60.4%) were diagnosed with B cell non-Hodgkin lymphoma (NHL), 164 (29.0%) were diagnosed with T cell NHL, and 60 (10.6%) were diagnosed with Hodgkin lymphoma. Among these patients, the PICC-UDEVT rates were 7.3%, 5.5% and 10.0%, respectively, and these rates were not significantly different (*P* = 0.494). A comparison of the lymphoma patients with the other cancer patients revealed differences in the patients' characteristics. The rate of PICC-UEDVT was higher in the lymphoma patients than in the other cancer patients (40/565, 7.1%; and 209/7463, 2.80%, respectively; *P* < 0.001) (Figure 1). A total of 346 of the lymphoma patients were younger than 52 years of age, and 219 were equal to or older than 52 years, and these numbers were 3854 and 3609, respectively, for the other cancer patients (*P* < 0.001). Therefore, the lymphoma patients were younger than the other cancer patients. The median platelet counts in the patients with lymphoma at their first visit and at the time of catheter insertion were 182 × 10^9^/L and 191 × 10^9^/L, respectively, and these numbers were 201 × 10^9^/L and 88 × 10^9^/L, respectively, in the other cancer patients, and these differences between the two patient groups were significant (*P* = 0.003 and *P* < 0.001, respectively). The treatments received also differed between the patients with lymphoma and those with other types of cancer. Radiotherapy was used less often in the lymphoma patients than in the other patients (18.2% vs. 55.8%, respectively; *P* < 0.001), whereas chemotherapy was used more often in the lymphoma patients (93.3% vs. 88.7%, respectively; *P* = 0.001) (Table 2). Logistic analysis revealed that a lymphoma diagnosis was a risk factor for UEDVT in the cancer patients inserted with PICC (OR: 3.849, 95% CI: 2.334–6.347, *P* < 0.001). Other patients' characteristics, as shown in Table 3, did not increase the odds of UEDVT (Table 3).

**Table 2:** Characteristics of cancer patients inserted with PICC stratified according to diagnosis

Characteristic	Lymphoma *n* = 565 (100%)	Others *n* = 7463 (100%)	*P*-value
Age (years)			
< 52	346 (61.2)	3854 (51.6)	< 0.001^b^
≥ 52	219 (38.8)	3609 (48.4)	
Gender			
Male	341 (60.4)	4430 (59.4)	NS^b^
Female	224 (39.6)	3033 (40.6)	
Radiotherapy			< 0.001^b^
Yes	100 (18.2)	3896 (55.8)	
No	465 (81.8)	3567 (54.2)	
Chemotherapy			0.001^b^
Yes	527 (93.3)	6622 (88.7)	
No	38 (6.7)	841 (11.3)	
Platelet at first visit	Median 182 × 10^9^/L	Median 201 × 10^9^/L	0.003^a^
Platelet at catheter insertion	Median 191 × 10^9^/L	Median 88 × 10^9^/L	< 0.001^a^

a Independent sample *t*-test

b Chi-square test

NS, not significant (*P* > 0.05)

PICC, peripherally inserted central venous catheter; UEDVT, upper extremity deep vein thrombosis.

**Table 3:** Logistic regression analysis of risk factors associated with UDEVT in cancer patients inserted with PICC (*n* = 8028)

Risk factor	Regression coefficient	Standard error	Odds ratio	95% Confidence interval	*P*-value
Age	−0.001	0.007	0.999	0.984–1.013	NS
Gender	0.015	0.205	1.015	0.679–1.517	NS
Lymphoma	1.348	0.255	3.849	2.334–6.347	< 0.001
Radiotherapy	−0.115	0.214	0.891	0.586–1.356	NS
Chemotherapy	0.365	0.400	1.440	0.658–3.151	NS
Platelet at first visit	−0.002	0.001	0.998	0.995–1.001	NS
Platelet at catheter insertion	0.000	0.001	1.000	0.998–1.003	NS

NS, not significant (*P* > 0.05)

PICC, peripherally inserted central venous catheter; UEDVT, upper extremity deep vein thrombosis.

## DISCUSSION

VTE commonly occurs in cancer patients, and the association between cancer and thrombosis has been known for many years [15]. This event may reflect the underlying biology of cancer because activation of the coagulation cascade and the generation of thrombin are often cited as mechanisms by which tumor propagation may occur [16]. Thrombotic events in cancer patients may be related to vascular access devices [7, 17]. Although the mechanisms by which catheter thrombosis develops are unclear, they may involve endothelial damage and local blood vortexing. In our study, 3.1% of the patients were diagnosed with PICC-UEDVT, which is consistent with the meta-analysis published by Vineet Chopra [6].

According to previous studies, the rate of PICC-UEDVT is between 1%–30% [3, 4, 18], whereas VTE occurs in approximately 0.1% of the general population annually [19]. A recent clinical trial has shown that many of these events remain clinically silent and that up to 75% of patients with a catheter are found to have thrombosis by ultrasound examination [20]. However, our results differ from these previous results. In our study, all of the patients were required to undergo ultrasound examination before removal of the catheter, which allowed us to identify patients with asymptomatic thrombosis. Diagnoses of thrombosis were confirmed by Doppler ultrasound, which is the standard diagnostic method. These examinations were performed after the emergence of symptoms (such as swelling in the upper extremities, pain, or leakage at the PICC site) or before removal of the catheter.

In our study, we identified differences between the patients with and without UEDVT in the history of radiotherapy, history of chemotherapy, and platelet counts at first visit and at the time of diagnosis. There was no significant difference in age between the thrombosis and non-thrombosis groups in our study. Age was reported to be an independent risk factor for thrombosis in some previous studies, although it was controversial [21, 22]. A comparison of the lymphoma patients with the other cancer patients revealed that age, history of radiotherapy, history of chemotherapy, and the platelet counts at first visit and at the time of catheter insertion were different. However, these factors had no statistically significant association with the UEDVT in the logistic regression analysis. Vascular endothelial cells produce increasing levels of procoagulants with advancing age, and this may be the reason behind the hypercoagulation [23]. The lymphoma patients were younger, which may have contributed to the age effect. Radiotherapy and chemotherapy tended to be positively correlated with thrombosis, perhaps because radiation of the neck or chest and chemotherapeutic agents contribute to the injury of the involved area and thereby increase the risk of thrombosis [24–27]. Another study has shown that cancer is associated with a 4.1-fold increased risk of thrombosis and that chemotherapy increases the risk by 6.5-fold [28]. Cancer and its treatments are therefore both risk factors; cancer patients have a VTE risk of 4%–20%, whereas this risk is 0.1% in the general population [29]. We were surprised to find that the platelet counts were lower at first visit in the thrombosis group, but the platelet counts at the time of catheter insertion were not significantly different between the thrombosis and non-thrombosis groups. All of the counts were within the normal range. This finding may have occurred because the thrombosis rate is not related to the platelet count [30, 31].

Many studies have established a strong link between VTE and cancer, but the link between VTE and lymphoma is less well documented. We conclude from our logistic analysis that a lymphoma diagnosis is associated with PICC-UEDVT. Other recent studies have also supported our hypothesis that lymphoma patients have a tendency to develop thrombosis. The rate of VTE events in patients with diffuse large B-cell lymphoma may be as high as 12.8% [32]. In our study, logistic analysis indicated that the patients with lymphoma were almost 4 times as likely to develop PICC-UEDVT as those with other types of malignancies. There were no significant differences in the rates of PICC-UDEVT among the patients with different subtypes of lymphoma.

In conclusion, the findings of our study indicate that patients with lymphoma may be predisposed to developing PICC-UEDVT compared with those with other types of malignancies. Identifying the mechanism underlying this relationship will require further study.

## MATERIALS AND METHODS

### Patients and study design

This retrospective study included consecutive patients who underwent PICC insertion between June 1st, 2007 and June 30th, 2015 at the Sun Yat-sen University Cancer Center (SYSUCC). Patient information was abstracted from electronic medical records. This study was performed in accordance with the Declaration of Helsinki, and it was approved by the Institutional Review Board (IRB) of the Sun Yat-Sen University Cancer Center.

This study recruited adult patients who met the following criteria. First, they were diagnosed with a malignancy and had complete medical data available, including age, sex, diagnosis, and medical history (including the history of radiotherapy before or chemotherapy after PICC insertion). Second, they had undergone routine blood testing (hemoglobin level and white blood cell and platelet counts) at their first visit and within 1 week of PICC insertion. Finally, data on PICC insertion and removal and the result of Doppler ultrasound performed before removal were recorded.

In the medical history, radiotherapy was defined as positive when the field of radiation included the chest or neck, and chemotherapy was defined as positive when the drugs were infused through the catheter. For patients who had more than one PICC insertion, only the first insertion was analyzed in our study. Patients with incomplete data due to PICC placement before hospital admission were excluded.

### PICC insertions

PICC insertions were prescribed by doctors and performed by a trained PICC nursing team at the Catheter Clinic of SYSUCC. Typically, patients underwent PICC insertion for the following reasons: the need for continuous intravenous infusion or special irritant drugs (such as anthracyclines or vinorelbine), poor vascular elasticity or small blood vessel diameters. The insertions were performed using a Standard Operating Procedure. A peripheral vein (the cephalic vein, basilic vein, median cubital vein, or brachial vein) was chosen by the nurse, and insertion was then performed using either ultrasound-guided puncture and cannulation or a blind technique (aiming toward a visible or palpable peripheral vein) until the tip rested in the distal superior vena cava or the cavo-atrial junction. The first-choice vein was the right basilic vein. The calibers of the catheters ranged from 4 to 6 French (most were close-ended, three-way valve PICCs manufactured by Bard (Bard Access Systems, Salt Lake City, UT, USA) and were made of silicone, single-lumen, 18-gauge catheters (4 Fr)) for adult patients, and their lengths ranged from 40 to 60 cm. Following insertion, the position of the PICC tip was verified by chest x-ray, and subsequent adjustments were performed according to the results.

### Follow-up

Demographic and background data, data related to PICC placement, and complications encountered during PICC placement were recorded by a nurse. The size of the catheter, length of the exposed part, arm circumference and conditions at the point of puncture were recorded before and after each use of the PICC. Then, the catheters were flushed every week in the catheter clinic and maintained by PICC nurses at either our hospital or other institutions.

PICC maintenance consisted of disinfection of the area around the PICC insertion site in addition to the insertion site itself and catheter, changing of the dressing and connector, and flushing and locking of the catheter with heparin saline. To disinfect the catheter, 75% alcohol was applied 3 times to clean the skin around it, and 2% chlorhexidine in 70% alcohol was applied 2 times to disinfect the whole site and the catheter. To flush and lock the catheter with heparin saline, 20 ml saline mixed with 200 U heparin was used before and after injection of medication, at the end of therapy, and every 7 days when the catheter was not in use.

In our hospital, patients require maintenance on the 2nd and 5th days after placement and subsequently once per week. The catheter line can be removed by a trained nurse after use, and ultrasound examination is required before it can be removed. Doppler ultrasound is recommended in the following situations: the appearance of symptoms of UEDVT (such as swelling in the upper extremities, pain, or leakage at the PICC site), clinical suspicion of thrombosis (even without symptoms), or diagnosis of deep vein thrombosis (DVT) at another site.

Doppler ultrasound was performed in the Ultrasound Department of SYSUCC using the most modern high-resolution, real-time, color Doppler ultrasound machines in the department, including a Sequoia 512, Philips IU22, GE LOGIQ E9 and TOSHIBA Aplio XG 790. The results were reported by a faculty member of one of the included departments (including 1 professor, 3 associate professors and 6 attending physicians).

### Diagnosis of thrombosis

PICC-associated thrombosis in the vein of insertion was diagnosed by Doppler ultrasound. Thrombosis was identified as positive when it included partial/complete thrombus in the deep veins of the arm, either inside or outside of the catheter.

### Statistical analysis

The following data were collected for statistical analyses: patient clinical information (age, gender, and cancer pathologic subtype), medical history (radiotherapy before PICC insertion and chemotherapy after PICC insertion), laboratory parameters at the first visit and within 1 week of PICC insertion (hemoglobin level and white blood cell and platelet counts), and whether thrombosis had occurred before removal of the catheter, as determined according to the Doppler ultrasound examination results. The patients' characteristics are presented as the median (range) or rate. The incidence of thrombosis was calculated as the total number of thrombosis events divided by the total number of PICCs placed (%). The independent sample *T* test was performed to compare the data (age and laboratory parameters). Data on gender, cancer pathologic subtypes, and medical history were analyzed using the Chi-square test. Logistic regression analysis was used to estimate unadjusted odds ratios (ORs) and 95% confidence intervals (CIs) of significant variables to identify the major risk factors for thrombosis. A 2-tailed *P*-value of < 0.05 was considered to indicate statistical significance. All statistical analyses were performed using SPSS version 22.0 for Windows.
